# Comparative effects of small intestinal glucose on blood pressure, heart rate, and noradrenaline responses in obese and healthy subjects

**DOI:** 10.14814/phy2.13610

**Published:** 2018-02-14

**Authors:** Laurence G. Trahair, Tongzhi Wu, Christine Feinle‐Bisset, Chinmay S. Marathe, Christopher K. Rayner, Michael Horowitz, Karen L. Jones

**Affiliations:** ^1^ School of Medicine University of Adelaide Adelaide South Australia Australia; ^2^ NHMRC Centre of Research Excellence in Translating Nutritional Science to Good Health Adelaide South Australia Australia; ^3^ Department of Gastroenterology and Hepatology Royal Adelaide Hospital Adelaide South Australia Australia; ^4^ Endocrine and Metabolic Unit Royal Adelaide Hospital Adelaide South Australia Australia

**Keywords:** Gastric emptying, intraduodenal, sympathetic

## Abstract

Meal consumption leads to an increase in sympathetic output to compensate for hemodynamic changes and maintain blood pressure (BP). Obesity is associated with a blunting of the sympathetic response to meal ingestion, but interpretation of studies investigating these responses is compromised by their failure to account for the rate of gastric emptying, which is an important determinant of postprandial cardiovascular and sympathetic responses and, in both health and obesity, exhibits a wide interindividual variation. We sought to determine the effects of intraduodenal glucose infusion, bypassing gastric emptying, on BP, heart rate (HR), and noradrenaline responses in obese and healthy control subjects. 12 obese subjects (age 36.6 ± 3.9 years, body mass index (BMI) 36.1 ± 1.3 kg/m^2^) and 23 controls (age 27.8 ± 2.4 years, BMI 22.4 ± 0.5 kg/m^2^) received intraduodenal infusions of glucose at 1 or 3 kcal/min, or saline, for 60 min (*t* = 0–60 min), followed by intraduodenal saline (*t* = 60–120 min). BP and HR were measured with an automatic cuff, and blood samples collected for measurement of plasma noradrenaline. Intraduodenal glucose at 1 kcal/min was associated with a fall in diastolic BP in the control subjects only (*P* < 0.01), with no change in systolic BP, HR or noradrenaline in either group. In both groups, intraduodenal glucose at 3 kcal/min was associated with a fall in diastolic (*P* < 0.01), but not systolic, BP, and rises in HR (*P* < 0.001) and plasma noradrenaline (*P* < 0.01), with no difference in responses between the groups. We conclude that cardiovascular and sympathetic responses to intraduodenal glucose infusion are comparable between obese and control subjects, and dependent on the rate of glucose delivery.

## Introduction

The postprandial cardiovascular response in health involves splanchnic blood pooling compensated for by increases in cardiac output and heart rate (HR), along with vasoconstriction in skeletal muscle and the peripheral vasculature, so there is minimal change in blood pressure (BP) (Sidery et al. 1991; Waaler and Eriksen 1992). These responses are driven by barostat activation and autonomic pathways (Kearney et al. 1995), and in healthy subjects, meal ingestion is associated with an increase in sympathetic nerve activity and plasma noradrenaline concentrations (Fagius and Berne 1994; Fagius et al. 1996).

Obesity is reportedly associated with a blunting of the sympathetic response (assessed by muscle sympathetic nerve activity (MSNA) and plasma noradrenaline) to ingestion of oral glucose (Vollenweider et al. 1994; Straznicky et al. 2009), particularly in individuals with insulin resistance and the metabolic syndrome (Spraul et al. 1994; Vollenweider et al. 1994; Straznicky et al. 2009). It has been suggested that impaired postprandial sympathetic responses may predispose to weight gain through a reduction in insulin‐induced vasodilation in skeletal muscle (Vollenweider et al. 1994). In contrast, the fasting sympathetic response has been reported to be elevated in obese subjects (Fagius 2003).

Interpretation of the outcomes of studies in obese subjects that have investigated the cardiovascular and sympathetic responses to ingested nutrients is confounded, because of failure to account for the rate of gastric emptying, which is now recognized to be of fundamental relevance (Trahair et al. 2012). Gastric emptying varies widely between individuals, but relatively little within an individual, so that in health, nutrients such as glucose usually empty from the stomach at an overall linear rate between 1 and 4 kcal/min (Collins et al. 1983). Gastric distension, even at modest volumes, increases sympathetic activity – the so‐called “gastrovascular reflex” (Rossi et al. 1998) – while intraduodenal administration of glucose, which “bypasses” the stomach and avoids gastric distension, at rates (2–3 kcal/min) within the normal physiological range increases plasma noradrenaline in healthy young adults (Trahair et al. 2012). In young subjects, intraduodenal infusion of glucose at 3 kcal/min is also associated with an increase in HR but little, if any, change in systolic BP (Trahair et al. 2012). In contrast, an identical glucose load in healthy older subjects reduces BP associated with a decreased plasma noradrenaline response (Trahair et al. 2012). More marked declines in BP occur in patients with postprandial hypotension (a fall in systolic BP >20 mmHg, sustained for at least 30 min, occurring within 2 h of a meal (Jansen and Lipsitz 1995)), an increasingly recognized disorder associated with relatively more rapid gastric emptying (Trahair et al. 2015). Accordingly, in healthy older subjects and people with postprandial hypotension, compensation for the postprandial rise in splanchnic blood flow is inadequate (Trahair et al. 2014).

These insights are of relevance to the interpretation of postprandial BP and sympathetic responses in the obese. In particular, gastric emptying has been reported to be accelerated substantially in a subset of obese subjects (Acosta et al. 2015), although observations have been inconsistent (Seimon et al. 2013), and there is a wide range of gastric emptying in the obese, as in healthy subjects (Vazquez Roque et al. 2006; Park et al. 2007; Seimon et al. 2013; Horner et al. 2015). Given that the rate of intraduodenal glucose delivery affects catecholamine responses in health (Trahair et al. 2012) variations in gastric emptying may be of relevance to postprandial catecholamine levels in the obese. There is, however, no information about the effect of gastric emptying on postprandial cardiovascular or noradrenaline responses in obese subjects.

The aim of this study was to determine the effects of intraduodenal glucose infusion at two rates within the normal range of gastric emptying on BP, HR and noradrenaline responses in obese and healthy control subjects. We hypothesized that HR and noradrenaline, but not BP, responses are dependent on the intraduodenal glucose load in both groups.

## Materials and Methods

### Subjects

Twelve obese subjects (2 female and 10 male, mean age 36.6 ± 3.9 years (range: 21–56 years), body mass index (BMI) 36.1 ± 1.3 kg/m^2^ (range: 31.1–44.9 kg/m^2^)), and 23 healthy control subjects (7 female and 16 male, mean age 27.8 ± 2.4 years (range: 19–54 years), BMI 22.4 ± 0.5 kg/m^2^ (range: 19.0–25.0 kg/m^2^)), were recruited by advertisement. Data in a subset of these subjects relating to blood glucose, serum insulin, and incretin hormone responses have been reported (Trahair et al. 2017). In all obese subjects, an oral glucose tolerance test (75 g glucose) was performed <6 weeks before the first intraduodenal study to exclude diabetes. All subjects were nonsmokers and none had a history of cardiovascular, hepatic, renal or gastrointestinal disease, epilepsy or alcohol abuse. No subject was pregnant, breast feeding, or taking medication known to influence BP or gastrointestinal function, and any other medication was withheld for 24 h prior to each study day.

### Protocol

Subjects were studied on three occasions, separated by at least 1 week. On each study day, the subject attended the Discipline of Medicine at the Royal Adelaide Hospital at 0830 h, after an overnight fast. Upon arrival at the laboratory, a silicone‐rubber, multilumen nasoduodenal catheter (external diameter ~4 mm) (Dentsleeve International, Mui Scientific, Mississauga, Canada) was inserted into the stomach via an anesthetized nostril and allowed to pass into the duodenum by peristalsis (Trahair et al. 2012). The catheter was designed so that, once positioned, the opening of an infusion channel (internal diameter ~1 mm) was located ~10 cm distal to the pylorus. Two other channels, with openings located in the antrum (2.5 cm proximal to the pylorus) and in the duodenum (2.5 cm distal to the pylorus), were perfused continuously with 0.9% saline (Trahair et al. 2012). The correct positioning of the catheter was maintained by continuous measurement of the transmucosal potential difference (TMPD) from the antral (−40 mV) and duodenal (0 mV) channels. This was achieved using a 0.9% saline‐filled reference electrode (20‐gauge intravenous (IV) cannula) that was inserted subcutaneously into the subject's forearm (Heddle et al. 1988; Trahair et al. 2012). After the catheter was positioned correctly, the subject was placed in a supine position and an IV cannula inserted into a left antecubital vein for blood sampling. The subject then remained in the recumbent position and was allowed to rest for 15–30 min.

Commencing at *t* = 0 min (~0930 h), each subject received an intraduodenal infusion of glucose at either 1 or 3 kcal/min (“G1” or “G3”, respectively), or 0.9% saline (“S”) for 60 min (*t* = 0–60 min), followed by saline for a further 60 min (*t* = 60–120 min). The order of the infusions on the three study days was randomized and studies were performed in a double‐blind fashion. All infusions were performed, using an automated volumetric infusion pump, at a rate of 5 mL/min (Imed Gemini PC‐1: IMED Corporation, San Diego, CA). At *t* = 120 min, the nasoduodenal catheter and cannulae were removed and the subject was offered a light meal prior to leaving the laboratory. On one of the study days, autonomic nerve function was evaluated following lunch using standardized cardiovascular reflex tests (Piha 1991).

The protocol was approved by the Research Ethics Committee of the Royal Adelaide Hospital, and each subject provided written, informed consent. All experiments were carried out in accordance with the Declaration of Helsinki.

### Measurements

#### Blood pressure and heart rate

BP and HR were measured using an automated oscillometric BP monitor (DINAMAP ProCare 100, GE Medical Systems, Milwaukee, WI), every 3 min during the rest period, and between *t* = 0–120 min. Baseline BP was calculated as an average of the three measurements obtained immediately prior to commencement of infusion (i.e., *t* = −9, *t* = −6 and *t* = −3 min) (Trahair et al. 2012).

#### Plasma noradrenaline

Venous blood samples were collected immediately prior to commencement of infusion (at *t* = −3 min) and then at 60 and 120 min for measurement of plasma noradrenaline (Trahair et al. 2012). Blood samples were obtained in tubes containing EDTA, centrifuged at 1490 *g* for 15 min and plasma separated and stored at −70°C for subsequent analysis. Noradrenaline was measured, using high performance liquid chromatography coupled with electrochemical detection (Waters Corporation, Milford, MA) (Holmes et al. 1994). The minimum detectable limit was 0.1 nmol/L and the intra‐assay coefficient of variation was 5.2%.

#### Autonomic nerve function

Autonomic nerve function was assessed using standardized cardiovascular reflex tests (Piha 1991). Parasympathetic function was evaluated by the variation (R‐R interval) of the heart rate during deep breathing and the response to standing (“30:15” ratio). Sympathetic function was assessed by the fall in systolic BP in response to standing. Each test result was scored according to age‐adjusted predefined criteria as 0 = normal, 1 = borderline and 2 = abnormal for a total maximum score of 6. A score ≥3 was considered to indicate autonomic dysfunction (Piha 1991).

#### Statistical analysis

Incremental areas under the curve (iAUCs) between *t* = 0–60 min were calculated for all variables, using the trapezoidal rule. For all variables, baseline values, iAUCs and changes over time between *t* = 0–60 min (i.e., time effect) were analyzed, using one‐way repeated measures ANOVA. Student's unpaired t‐test was used to compare iAUCs between the obese and control subjects. All analyses were performed, using SPSS 17.0.0 (SPSS Inc, Chicago, IL). Data are presented as mean values ± standard error of the mean (SEM). A *P* value <0.05 was considered significant.

## Results

The studies were well tolerated, three subjects in the control group experienced mild symptoms consistent with hypoglycemia which did not require intervention. Baseline values are listed in Table 1. There was a trend for the obese subjects to be older than the controls (*P* = 0.05). None of the obese subjects had cardiovascular autonomic neuropathy (score 1.8 ± 0.3).

**Table 1:** Baseline variables prior to each treatment in control (*n* = 23) and obese (*n* = 12) subjects

	Control subjects				Obese subjects				Controls versus obese (baseline difference for all 3 conditions)
	Saline	1 kcal/min	3 kcal/min	*P* value (differences at baseline)	Saline	1 kcal/min	3 kcal/min	*P* value (differences at baseline)	
Systolic BP (mmHg)	111.6 ± 2.1	111.4 ± 2.3	112.1 ± 2.1	*P* = 0.82	123.5 ± 3.3	122.3 ± 3.5	123.3 ± 2.9	*P* = 0.83	*P* < 0.01
Diastolic BP (mmHg)	63.7 ± 1.6	63.1 ± 1.4	63.9 ± 1.3	*P* = 0.72	71.6 ± 2.3	71.3 ± 3.4	69.9 ± 2.9	*P* = 0.27	*P* < 0.01
Heart rate (BPM)	57.5 ± 1.4	58.5 ± 1.4	58.4 ± 1.6	*P* = 0.52	60.8 ± 2.6	60.4 ± 2.6	59.3 ± 2.5	*P* = 0.50	*P* = 0.44
Plasma noradrenaline (nmol/L)	1.23 ± 0.19	1.24 ± 0.18	1.20 ± 0.15	*P* = 0.94	0.75 ± 0.11	0.79 ± 0.09	0.83 ± 0.13	*P* = 0.79	*P* = 0.09

All values are mean ± SEM. BP; blood pressure, BPM; beats per minute.

### Systolic blood pressure

#### Controls

**Figure 1:**
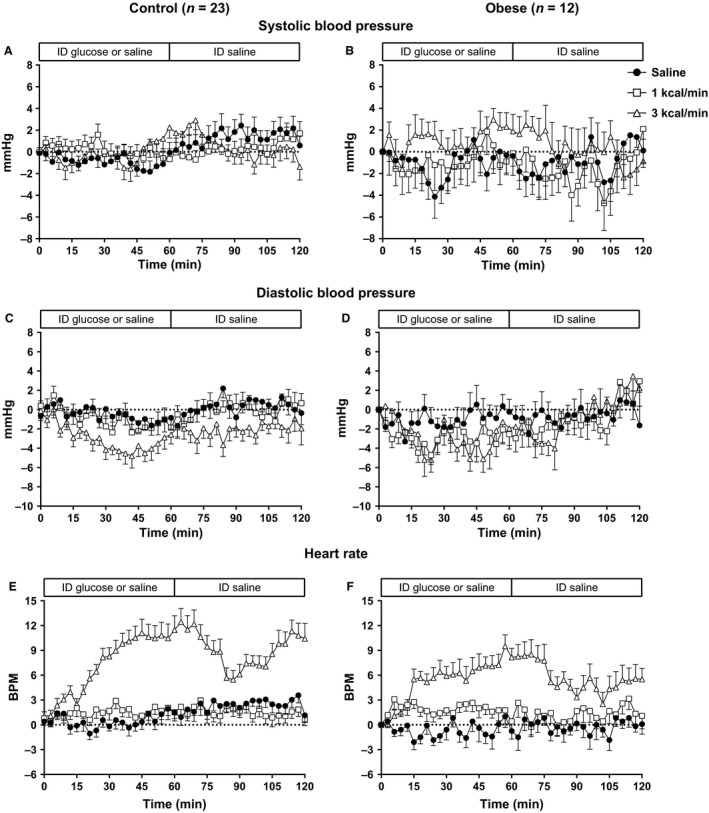
Systolic (A, B), diastolic (C, D) blood pressure (BP) and heart rate (E, F) responses to intraduodenal infusion of saline, or glucose at 1 kcal/min (G1) or 3 kcal/min (G3) between *t* = 0–60 min, followed by intraduodenal saline between *t* = 60–120 min, in healthy control (A, C, E) (*n* = 23) or obese (B, D, F) (*n* = 12) subjects. In the control group there is a fall in diastolic BP during G1 (*P* < 0.01) and G3 (*P* < 0.001) and a rise in heart rate during G3 (*P* < 0.001). In the obese subjects, there is a fall in diastolic BP (*P* < 0.01) and a rise in heart rate (*P* < 0.001) during G3. Responses did not differ between the two groups.

There was no difference in systolic BP at baseline (*P* = 0.82), nor any change in systolic BP over time, during any of the conditions (S: *P* = 0.74, G1: *P* = 0.82, G3: *P* = 0.10) (Fig. 1A). There was no treatment effect on the iAUCs between the conditions (*P* = 0.48).

#### Obese

There was no difference in systolic BP at baseline (*P* = 0.83), nor any change in systolic BP over time, during any of the conditions (S: *P* = 0.27, G1: *P* = 0.94, G3: *P* = 0.63) (Fig. 1B). There was no treatment effect on the iAUCs between the conditions (*P* = 0.37).

#### Between‐group comparisons

Baseline systolic BP was greater in the obese group on all three study conditions (*P* < 0.05 for all). There was no difference between the groups in the systolic BP response during any of the conditions (S: *P* = 0.76, G1: *P* = 0.46, G3: *P* = 0.32).

### Diastolic blood pressure

#### Controls

There was no difference in diastolic BP at baseline between the three conditions (*P* = 0.72). There was no change in diastolic BP over time during S (*P* = 0.49), however, there was a fall during both G1 (*P* < 0.01) and G3 (*P* < 0.001) (Fig. 1C). There was a treatment effect on the iAUCs between the conditions (*P* < 0.05), so that the iAUC for G3 was less than S (*P* < 0.05) and G1 (*P* < 0.05), with no difference between G1 and S (*P* = 1.00).

#### Obese

There was no difference in diastolic BP at baseline between the three conditions (*P* = 0.27). There was no change in diastolic BP over time during S (*P* = 0.72) or G1 (*P* = 0.35), however, there was a fall during G3 (*P* < 0.01) (Fig. 1D). There was no treatment effect on the iAUCs between the conditions (*P* = 0.24).

#### Between‐group comparisons

Baseline diastolic BP was greater in the obese group on all three study conditions (*P* < 0.05 for all). There was no difference between the groups in the diastolic BP response during any of the conditions (S: *P* = 0.62, G1: *P* = 0.08, G3: *P* = 0.89).

### Heart rate

#### Controls

There was no difference in HR at baseline between the three conditions (*P* = 0.52). There was no change in HR over time during S (*P* = 0.14) or G1 (*P* = 0.41), but there was a prompt and sustained increase during G3 (*P* < 0.001) (Fig. 1E). There was a treatment effect on the iAUCs between the conditions (*P* < 0.001), so that the iAUC for G3 was greater than S (*P* < 0.001) and G1 (*P* < 0.001), with no difference between G1 and S (*P* = 0.38).

#### Obese

There was no difference in HR at baseline between the three conditions (*P* = 0.50). There was no change in HR over time during S (*P* = 0.70) or G1 (*P* = 0.83), but there was a sustained increase during G3 (*P* < 0.001) (Fig. 1F). There was a treatment effect on the iAUCs between the conditions (*P* < 0.001), so that the iAUC for G3 was greater than S (*P* < 0.001) and G1 (*P* < 0.05), with no difference between G1 and S (*P* = 0.10).

#### Between‐group comparisons

There was no difference in HR between the groups, either at baseline (*P* = 0.44), or in response to any of the conditions (S: *P* = 0.33, G1: *P* = 0.72, G3: *P* = 0.20).

### Plasma noradrenaline

#### Control

**Figure 2:**
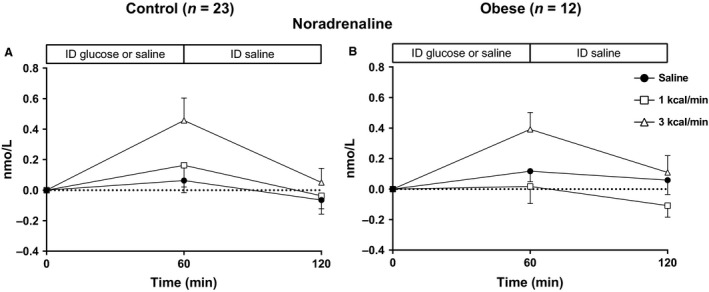
Plasma noradrenaline responses to intraduodenal infusion of saline, or glucose at 1 kcal/min (G1) or 3 kcal/min (G3) between *t* = 0–60 min, followed by intraduodenal saline between *t* = 60–120 min, in healthy control (A) (*n* = 23) or obese (B) (*n* = 12) subjects. In both groups, there was a rise in noradrenaline during G3 (*P* < 0.01) without any difference in the response between the two groups.

There was no difference in noradrenaline at baseline between the three conditions (*P* = 0.94). There was no change in noradrenaline during S (*P* = 0.41) or G1 (*P* = 0.33), but there was a rise during G3 (*P* < 0.01) (Fig. 2A). There was a trend for a treatment effect on the iAUCs between the conditions (*P* = 0.07).

#### Obese

There was no difference in noradrenaline at baseline between the three conditions (*P* = 0.79). There was no change in noradrenaline during S (*P* = 0.35) or G1 (*P* = 0.35), but there was a rise during G3 (*P* < 0.001) (Fig. 2B). There was a treatment effect on the iAUCs between the conditions (*P* < 0.05), with no individual differences between S and G1 (*P* = 1.00), G3 (*P* = 0.21), or between G1 and G3 (*P* = 0.11).

#### Between‐group comparisons

There was no difference in noradrenaline between the groups, either at baseline (*P* = 0.09), or in response to any of the conditions (S: *P* = 0.71, G1: *P* = 0.56, G3: *P* = 0.75).

## Discussion

Our study has evaluated the effects of intraduodenal glucose infusion at two rates within the physiological range of gastric emptying on BP, HR and plasma noradrenaline in obese adults and compared these responses to those in healthy lean subjects. Novel observations are that (i) neither glucose load affected systolic BP in either group and (ii) intraduodenal glucose at 3 kcal/min, but not at 1 kcal/min, was associated with a comparable modest decline in diastolic BP and increases in HR and plasma noradrenaline in both groups. Predictably, baseline systolic and diastolic BP were slightly higher in the obese. These observations suggest that in “younger” adult subjects with and without obesity, postprandial BP and sympathetic responses are dependent on the rate of gastric emptying.

Studies of gastric emptying in obesity are numerous given the relevance to appetite regulation and postprandial glycaemic control (Vazquez Roque et al. 2006; Park et al. 2007; Seimon et al. 2013; Horner et al. 2015). Gastric emptying in the obese has been variously reported to be faster (Vazquez Roque et al. 2006; Acosta et al. 2015; Horner et al. 2015), slower (Horowitz et al. 1983), or comparable (Park et al. 2007; Seimon et al. 2013) to healthy subjects. Despite these inconsistencies, which may, in part, reflect prior patterns of macronutrient intake, it is clear that the inter‐individual variation in gastric emptying in the obese is large, as is the case in healthy subjects, and that gastric emptying is accelerated substantially in some (Acosta et al. 2015). To our knowledge, no studies have evaluated the sympathetic response to gastric distension in the obese; which is known to be diminished in older subjects (van Orshoven et al. 2004). Our observations relating to the noradrenaline responses to intraduodenal glucose in the younger subjects are consistent with our previous studies (Trahair et al. 2012). It is now clear that these responses are also dependent on the duodenal glucose load in the obese, as well as comparable between the groups. We anticipated that there would be no fall in systolic BP, and a rise in HR in response to the 3 kcal/min duodenal glucose load in the healthy subjects (Trahair et al. 2012), and this proved to be the case, with comparable responses in the obese. In both groups, there was a modest decline in diastolic BP. This is not unexpected as diastolic BP is dependent on peripheral vascular resistance, while systolic BP is more dependent on preload and contractility, both of which would be reduced with inadequate increase in sympathetic output (Guyenet 2006).

Some limitations of our study should be appreciated. Our study utilized a nonphysiological test “meal”, to eliminate factors relating to gastric emptying. The number of subjects was not large, but the observations appeared clear‐cut. While there was a wide range of subjects studied, and the obese subjects tended to be older than the controls, the difference was only minor and is unlikely to affect postprandial noradrenaline responses (Trahair et al. 2012). The obese subjects were not categorized according to insulin resistance. Plasma noradrenaline was only assessed at one time point after the intraduodenal glucose, given the exploratory nature of the study, but the timing is likely to represent maximal response given the study design. Noradrenaline concentrations in the forearm circulation are, of course, primarily an index of sympathetic activity in the forearm musculature, but are generally regarded as a useful measure of average sympathetic output (Goldstein et al. 1983). Finally, we did not measure the splanchnic or skeletal muscle blood flow responses.

In conclusion, the cardiovascular and sympathetic responses to intraduodenal glucose infusion at two rates within the normal range of gastric emptying are comparable in obese subjects and healthy lean controls. Since the responses were dependent on the rate of intraduodenal glucose infusion, future studies investigating these effects should take into account the rate of gastric emptying.

## Conflict of Interest

MH has participated in the advisory boards and/or symposia for Novo Nordisk, Sanofi, Novartis, Eli Lilly, Merck Sharp & Dohme, Boehringer Ingelheim, and AstraZeneca and has received honoraria for this activity. CR has received research funding from Sanofi, Novartis, Eli Lilly, Merck Sharp & Dohme, and AstraZeneca. KLJ has received research funding from Sanofi, Merck Sharp & Dohme, and AstraZeneca. None of the other authors has any personal or financial conflict of interest to declare.
